# ‘Mentoring the Gap’ for Junior Doctors: Promoting an under-utilised resource back to centre-stage

**DOI:** 10.15694/mep.2018.0000278.1

**Published:** 2018-12-11

**Authors:** Lucy Havard, Tom Baker

**Affiliations:** 1 University College London; 2 Royal College of Physicians

**Keywords:** Mentoring, medical education, near-peer learning, professional development, post-graduate education, peer support

## Abstract

This article was migrated. The article was marked as recommended.

Mentoring in medicine is not a new concept. However in recent years it has been sadly neglected and consigned to the wings of the medical education theatre. In this age of disparate clinical teams, disillusionment and shift working, the NHS needs to proactively nurture and develop junior doctors to support them on their career path. In this paper, we argue that effective mentoring is key to achieving this goal.

*Context and methods*: The researcher is a Core Medical Trainee (CMT) and set up a near-peer mentoring programme between Foundation Year (FY) doctors and CMTs. A focus group was conducted and inductive thematic analysis was used to analyse the resulting transcript. Thematic maps demonstrating the benefits and barriers to mentoring were produced.

*Results*: Results demonstrated that mentoring was useful for mentees’ personal development in terms of networking opportunities, pastoral support and the sharing of experiences. Positivity and camaraderie were key to an effective mentoring partnership, whilst rota clashes posed a significant barrier. Dangers identified included mentors adhering too tightly to a ‘mentoring formula’ and encouraging mentees to become ‘carbon copies’ of themselves.

*Conclusions*: This study has provided a valuable insight into the benefits of mentoring for junior doctors. Recommendations include promotion and active creation of formal mentoring programmes, and integration of formal mentoring training into the CMT curriculum.

## Introduction

Mentoring is not a new concept. Indeed, it has been a key component of a medical education from the advent of the profession and the days of apprenticeship ( Platz & Hyman, 2013). The origins of mentoring are rooted in the depths of antiquity; The Greats of the history of medicine: Hippocrates, Galen, Jenner, Hunter, Freud, Pasteur - all were mentored and were mentors ( Conrad et al, 2009).

The traditional concept of mentorship is based on a hierarchical dyadic relationship whereby “a senior faculty member counsels and instructs a junior protégé” ( Johnson et al, 2011, pp.1578). Regretfully, over the past few decades, mentorship has taken a back seat in medical education. This is despite mentoring being prominent in other fields, such as business and law, and, closer to home, in the nursing profession ( Bussey-Jones et al, 2006). In medicine, there is no longer the firm structure that helped nurture natural mentoring relationships. Instead, the increased emphasis on shift work and shorter rotations for junior doctors has been detrimental to the traditional model of mentoring, squeezing it out of the working environment. Understandably, senior registrars are less inclined to invest time and effort in mentoring juniors if they are only working together for a short while ( Platz & Hyman, 2013). However, in recent years, some effort has been made to overcome such obstacles and new mentorship models have been developed to try and improve access to mentoring.

Mentoring is a topic that has enjoyed increasing attention in the medical education sphere recently ( Platz & Hyman, 2013). The vast majority of the literature focuses on mentoring in the field of academic (as opposed to clinical) medicine; mentoring in the discipline of surgery has also been explored to some extent. However, mentoring within general medicine has been comparatively neglected ( Lord et al, 2012, pp.378).

In today’s economic climate, with the NHS stretched to breaking point, mentoring could provide a strategy for building morale, increasing retention, in a financially favourable manner. Although training of mentors admittedly requires some resources, we strongly believe that this investment pays handsome dividends.

A literature review was conducted in the preliminary stage of this project, which demonstrated the value of mentoring in career and personal development. Although some disadvantages were acknowledged, the vast majority of the literature supports mentoring as an important development tool. There is limited literature focusing on the mentor perspective and comparing formal and informal mentoring programmes within medicine. In attempting to address this gap in the literature, this paper will address the following question: ‘ *What are the perceived benefits of near-peer (NP) mentoring for Foundation Year (FY) doctors?*’

In answering the research question proposed, the following sub-questions will be explored:

• What are the benefits and challenges of formal versus informal mentoring?
• How does the mentoring format (traditional dyadic mentoring; near-peer mentoring; peer mentoring) impact upon the mentoring relationship?
• How accessible is mentoring to junior doctors currently?

## Methods

The researcher is a second year CMT and set up a mentoring programme between FY doctors and CMTs in September 2015. The research project took place at a busy District General Hospital (DGH) where there are 88 FY doctors and 34 CMTs. The study population was made up of the 29 FY doctors who adopted the role of mentee in the mentoring programme.

All 29 FY mentees were invited to participate in the focus group via email. A total of four to eight focus group participants was aimed for, and a variety of times and locations were offered to maximise participation.

A Bricolage approach to analysis was employed to generate meaning and draw conclusions. As Kvale (2009, p.119) explains, this approach “goes beyond following specific techniques or approaches to interview analysis and draws in a variety of techniques and theoretical concepts”. Content analysis was used to a certain degree, as was meaning interpretation. Detailed discourse analysis was also engaged, particularly with emotive areas of speech. Inductive thematic analysis was the primary means of analysis; a version of Braun and Clarke’s ‘Six phases of thematic analysis’ ( 2006), depicted below, was used to help guide this process:

**Figure 1.**
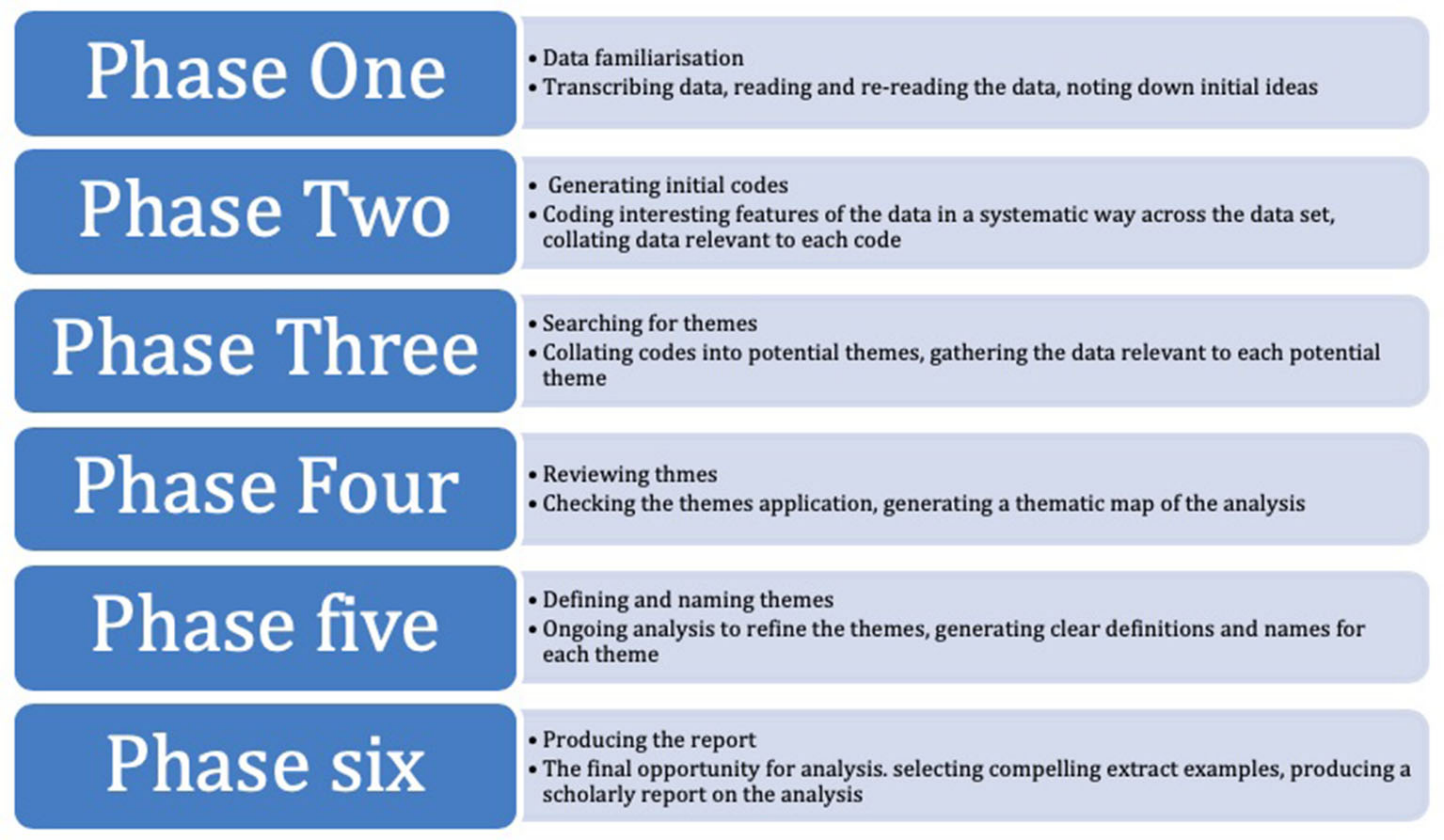
Six phases of thematic analysis (adapted from Braun and Clarke, 2006)

The researcher transcribed the audio recording of the focus group which conferred familiarity with the data and in itself resembled the initial stage of analysis. The resulting transcript was read and re-read several times and notes were made on potential codes and particularly interesting discussion points made in the focus group. Analysis was *recursive* ( Braun & Clarke, 2006), and the researcher went backwards and forwards between the transcript and original audio recording, developing codes as needed.

Although there were some pre-formed themes that had been identified from the initial literature review, the themes were largely emergent from the data collected from the focus group. Multiple mind-maps were generated for each sub-question, and the themes and codes identified were condensed further and further through multiple revisions until a key conceptual framework remained. At the end of this process, the themes were reviewed and the initial transcript and audio recording revisited in order to check that the themes identified were an accurate representation of the data set. A final thematic map was then produced to enable clear presentation.

### Reliability and validity

This study achieves a reasonable degree of validity but a limited level of reliability. A focus group discussion is shaped by the participants, their interactions and the focus group dynamic. Clearly, if the focus group were run again with another set of mentees from the same cohort, the transcript would be different, compromising reliability. However, the researcher would argue that similar themes would likely evolve. The researcher attempted to improve the reliability of the transcription process by revisiting both the typed transcript, *and* the recorded focus group when analysing the data. This was to ensure that the conclusions drawn were representative of the original focus group discussion.

Regarding validity, a focus group was an appropriate qualitative method to explore the research question posed and gain a deeper understanding of mentoring from the mentee perspective. The researcher had prior experience of running focus groups and had been educated in this field; both these factors acted to improve construct validity. External validity was potentially compromised through the possibility of self-selection bias. All the focus group participants volunteered, possibly because they had a positive experience of mentoring. However, it is also possible that the offer of a focus group might have induced those who did not have a positive experience to make their feelings known. Self-selection bias is certainly possible and a threat to external validity, although it is unclear whether this would impact upon the results in a positive or negative way.

### Ethics

Regarding the ethics of this study, a full ethics review was completed by the ethics committee at University College London (UCL) in line with the British Educational Research Association ethical guidelines for educational research ( BERA, 2011). Patients were not put at risk in the research project and no patient-sensitive data was collected. There was no personal information collected regarding participants’ health, mental health or high stakes assessment performance. It was deemed that there would not be a negative impact of this research on the institution in question as absolute anonymity of data was maintained.

## Results/Analysis

The final focus group comprised five mentees from the mentoring programme, all female, aged between 24 and 27. Four of the five mentees had experienced some form of mentoring prior to enrolling in the programme. The focus group participants had all met each other in the working environment prior to the focus group; it was hoped that this would make for a relaxed focus group dynamic. The focus group lasted 50 minutes.

The pre-formed themes were generated from the literature review. There were 14 themes identified which broadly fit into one of the four following overarching themes: prevalence of mentoring; barriers to mentoring; requirements for effective mentoring; and current thoughts regarding the state of mentoring today.

Generally, the focus group found the mentoring scheme to be beneficial and useful for their personal development. One participant had not had a successful mentoring relationship with the allocated mentor as they had been unable to find a mutually appropriate time to meet: rota clashes were described as one of the significant barriers to effective mentoring. Despite this, the mentee had experienced informal mentoring which had been useful.

The key attributes of successful mentors were discussed, as were the strengths and weaknesses of informal and formal mentoring schemes. The desire and need for mentoring for junior doctors was reiterated on several occasions, and described as particularly pertinent given the unsettlement regarding the 2016 junior doctor contract ( NHS Employers, 2016) and the threat of industrial action.

The focus group recognised a growing culture for mentoring. They identified improved accessibility secondary to the use of various modes of mentoring (peer, near-peer, dyadic, virtual, and self-mentoring). Formal training for mentors gave those junior doctors the tools needed to identify potential mentors for themselves, whilst the precipitation of a ‘mentoring culture’ meant that mentees were also encouraged to become mentors. However, there was still a call for increased availability of formal mentoring programmes. Although the existence of informal mentoring was recognised, the need for the correct ‘mentoring formula’ with engagement, enthusiasm, and compatibility between both mentor and mentee was noted in order to secure successful mentoring partnerships.

**Figure 2.**
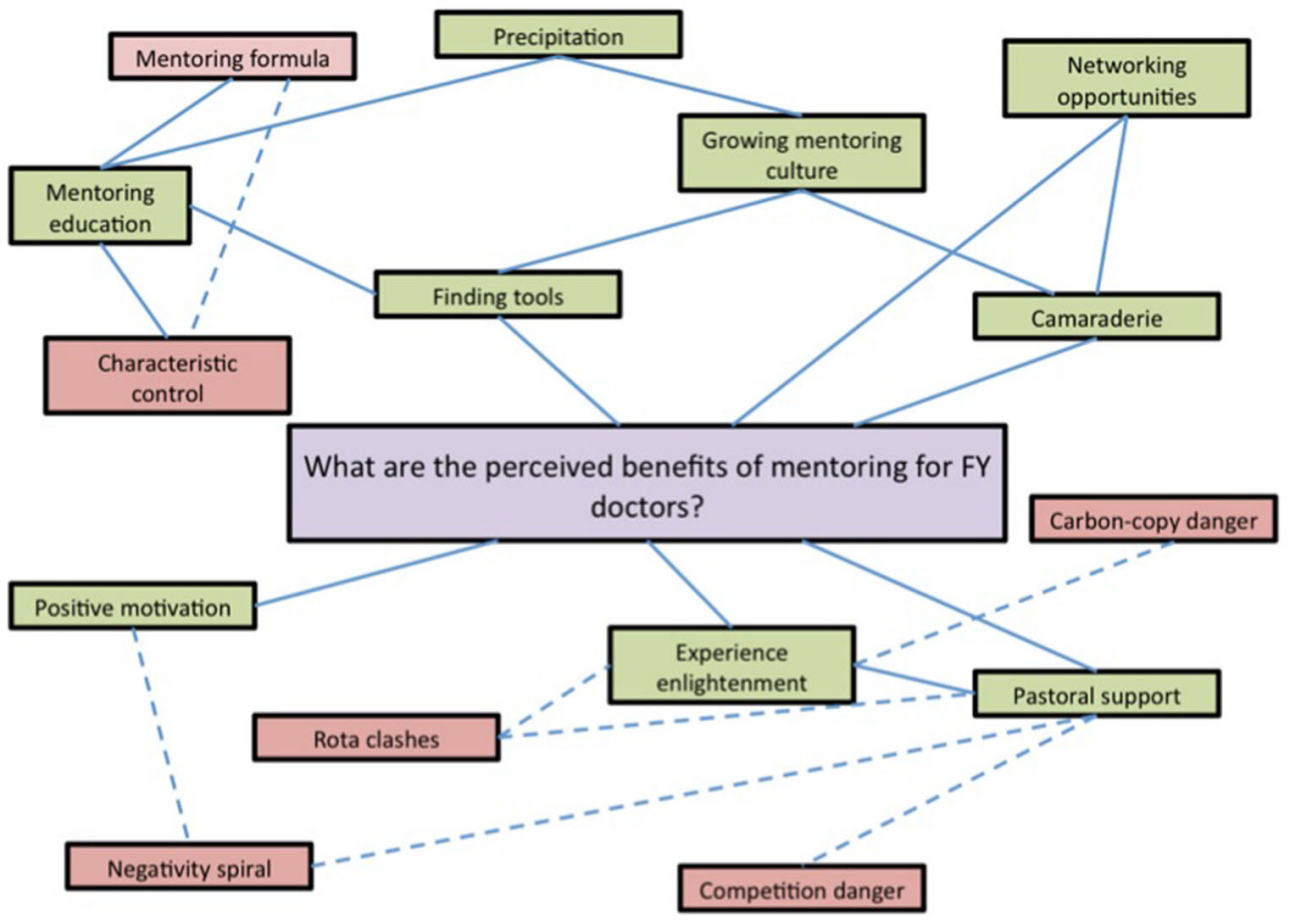
Condensed thematic map

A condensed thematic map with the key findings from the study was produced ( figure 2). The positive aspects of mentoring are highlighted in green whilst the negative aspects are highlighted in red for clear presentation. Solid lines represent supporting related themes whilst dashed lines represent directly contradictory or opposing themes.

## Discussion

The benefits and challenges of formal and informal mentoring generated lively focus group discussion. There were advantages and disadvantages identified for each, with rota issues a significant barrier to effective formal mentoring. The overall preference in the focus group was for informal mentoring. This was deemed to be even more effective when the mentor had access to ‘mentoring education’ - essentially, instruction in how to mentor effectively. However, this training was typically available only with formalmentoring programmes. A combination of formal and informal mentoring was therefore deemed to be most effective.

The focus group afforded greater prominence to the presence of positivity and camaraderie in the mentoring partnership as opposed to highlighting a particularly effective mode of mentoring (dyadic, near-peer or peer mentoring). Peer mentors were deemed to be particularly approachable and helpful for the purposes of networking. However, the potentially limited experience of a peer was also acknowledged.

Informal mentoring was judged to be infinitely accessible, especially in the provision of role models. Formal mentoring was less accessible, although the idea of virtual mentoring was suggested as a potential solution to this. The concept of precipitationwas introduced as helping to amplify the accessibility of mentoring for junior doctors.

### Limitations of the study

The small study size must be acknowledged. Although the target of between four and eight focus group participants was met, the reliability of the data may have been improved through running multiple focus groups, with cross-referencing between data sets to improve both validity and reliability. The potential for self-selection bias must be considered, as interestingly, the focus group participants were all female. This was not representative of the study participants where only 57% (17 females out of a total of 29 mentees) were female. This limits the generalisability of results. It would be interesting to see whether male participation in the focus group would have changed the focus group dynamic and hence the issues discussed and opinions presented. The question as to why no males volunteered to participate in the focus group also invites further investigation: would they have been more inclined to participate in a questionnaire rather than a focus group? Or was it just by chance that no males were available on the dates suggested for the study? Perhaps they did not find mentoring useful and therefore did not want to participate in the focus group? This is all supposition, but further work would certainly involve investigating the views of males on the mentoring programme.

The issue of researcher-related bias has already been considered. One approach to tackle this would be for the researcher to use a reflexive journal, which ‘sensitises the interviewer to his or her prejudices and subjectivities, while more fully informing the researcher of the impact of these influences on the credibility of the research outcomes’ ( Roller, 2012, p.4). This would be a valuable technique for use in future studies.

Given the phenomenological nature of this study, it has limited generalisability. The study took place at one particular hospital in relation to one specific mentoring scheme over the course of one year. It would be interesting to see whether similar results would be achieved in subsequent years with different cohorts of mentors and mentees. Despite this, the findings of this study are extremely significant for the mentoring scheme in question and the conclusions are still worth considering within the wider context of mentoring for junior doctors.

## Conclusion

In conclusion, the focus group considered their mentoring experience to be mostly positive. Although some challenges were discussed in terms of initiating mentoring, all focus group participants felt they had benefitted from formal mentoring, informal mentoring, or a combination of these.

The greatest pitfall voiced in the focus group was to be wary of a ‘carbon-copy danger’: essentially mentors avoiding encouraging mentees to just follow in their own footsteps. Mentoring was acknowledged as a very individual process with mentors and mentees having varying educational and development needs.

The current availability of mentoring was deemed insufficient for junior doctors. There was a call for more formal mentoring programmes with the hope that this would encourage a growing mentoring culture with the subsequent precipitation of more informal mentoring.

Positive motivation was judged essential to a successful mentoring partnership. A mentoring education was considered useful for mentors in their mentoring role and additionally in providing them with the ‘finding tools’ to identify their own mentors, helping promote a ‘bidirectional mentoring cycle’.

This paper has sought to demonstrate the wide-ranging benefits of mentoring for junior doctors, as well as some of the potential pitfalls to avoid. Given our findings, we would suggest promotion and active creation of formal mentoring programmes, integration of formal mentoring training into the CMT curriculum, and improved access to resources to facilitate self-mentoring and virtual mentoring.

## Take Home Messages

• A ‘mentor’ is an experienced, empathetic person who guides another individual (the mentee) through their personal and professional development ( SCOPME, 1998).
• Mentoring is currently an underutilised resource, despite its potential to benefit junior doctors’ personal and professional development.
• Barriers to effective formal mentoring include incompatible rotas whilst dangers include encouraging the creation of a ‘carbon copy’ of the mentor.
• Formal mentoring programmes with appropriate mentor training can precipitate informal mentoring which can be particularly beneficial.

## Notes On Contributors

Dr Lucy Havard completed a Masters in Medical Education in September 2017. She is now working as a Medical Registrar having completed Core Medical Training in London. Her medical education interests comprise mentoring and near-peer teaching.

Mr Tom Baker Completed a Masters in Teaching and Learning in 2013. He is now working as Head of Education Programmes at the Royal College of Physicians of London. He is also programme director for the RCP/UCL MSc in Medical Education. His interests are mentoring, assessment, leadership, and curriculum design.

## Appendices

### Appendix 1: Focus group schedule

• Do you feel you benefitted from the mentoring programme? If so, how?
• What do you feel are the qualities of a good mentor?
• Do you feel it is better to have a structured mentoring programme or concentrate more on informal mentoring in the clinical environment?
• How have you found being mentored by someone just a couple of years more senior than yourself? Do you think it’s better to have a more senior mentor? Have you had any experience of peer mentoring and if so, was this helpful?
• How accessible do you feel mentoring is to junior doctors currently?
• What do you think the role of mentoring is for junior doctors in today’s clinical environment? Does it actually have a role currently?
