# Supplementary material for: ‘Mentoring the Gap’ for Junior Doctors: Promoting an under-utilised resource back to centre-stage
Source: MedEdPublish (2016). 2018 Dec 11;7:278. [Version 1] doi: 10.15694/mep.2018.0000278.1 (PMC10711952; doi:10.15694/mep.2018.0000278.1)
Supplement: Peer review history

# Reviewer report: Reviewer response for version 1

**DOI:** 10.21956/mep.19725.r29543

**Published:** 2019-06-10

**Authors:** Trevor Gibbs

**Affiliations:** 1 AMEE

This review has been migrated. The reviewer awarded 4 stars out of 5 As someone who has a very close educational relationship to junior doctors and their development, I enjoyed reading this well written and researched paper. many of the authors findings from their research and their small study are not surprising , but the clarity that they have presented the does highlight the difficulties experienced by the junior doctors, which in turn, I feel, do affect quality of care.I will not expand on my co-reviewers excellent comments, many of which I agree with. Only to suggest that this paper is written by all involved in the mentoring of our junior colleagues.

Reviewer Expertise:

NA

No decision status is available

# Reviewer report: Reviewer response for version 1

**DOI:** 10.21956/mep.19725.r29542

**Published:** 2018-12-20

**Authors:** Megan Anakin

**Affiliations:** 1 University of Otago

This review has been migrated. The reviewer awarded 3 stars out of 5 Thank you for inviting me to review this article. It is good to see an article investigating the topic of mentoring from the student perspective. At my medical school, we have a long history of using formal mentoring groups with our undergraduate medical students and we are in the process of evaluating their functioning and outcomes. Therefore, this article is a timely read that I will share with my colleagues.While reading this article, I had several thoughts about how it might be improved. The authors may wish to follow up on the points below or consider them for when they construct their next article for publication.Please consider omitting the abbreviations CMT and FY in such a short article. It increases the reading difficulty for the reader. Please do not assume that an international reader is familiar with the names and functions of ‘Core Medical Trainee’ and ‘foundation year doctor’ roles in the UK medical profession. For example, in my country, we do not used the same designations, therefore, I was wondering who is more senior? The reader had to make an inference when the role of foundation year doctor was described in the study as a mentee.In the introduction the authors state that they have performed a literature review. It would be helpful to know more the limited literature found about mentoring. This enhancement would further strengthen the warrant for the study.In the methods section, the bricolage approach was well described so that another researcher could conduct a literature review and arrive at similar conclusions, however, the themes generated from the literature review and later used guided the analysis of the data are not stated explicitly in this section. For a complete description of the data collection, the authors might consider including the questions that were asked to the focus group. I’m assuming that the focus group questions were related to the three sub-questions presented at the end of the introduction section.Since this is a qualitative study, I am wondering about the use of the terms reliability and validity. With qualitative methods, these concepts often manifest themselves in how clearly the researcher explains and justifies the steps taken to design the study and to collect and interpret the data. These features are often described in terms such as credibility, trustworthiness, representativeness, and robustness. Running the focus group again with a different set of mentees might result in different themes emerging if the composition of the group was not selected to consist of people that represent the different possible views held by individuals in the cohort. As noted by the authors in the discussion section, this difference is often why more than one focus group is conducted. In this study, the researchers may be looking for ‘saturation’, when no new themes emerge with new data being collected (see Denzin, NK, Lincoln YS. 2005. The Sage handbook of qualitative research. Thousand Oaks (CA): Sage Publications Ltd). Please consider adding a reference to Appendix 1 in the methods section. I found the heading “Results/Analysis” a little confusing. Please consider describing the methods used to conduct the data analysis before the results are presented. In the results section, I am wondering if the pre-formed themes could have been introduced to the reader earlier. The results section might be strengthened if the themes that emerged from the analysis were reported with representative quotations to support the interpretation of the data. In the discussion section, please consider revising the following sentence: “There were advantages and disadvantages identified for each (Is a noun missing here?), with rota issues a significant barrier to effective formal mentoring”. In the limitations of the study section, the generalisability of the study’s findings is discussed twice. The authors might consider consolidating the issues about generalisability or perhaps focusing this part of the discussion on how the findings were representative of the cohort that was sampled. In the conclusion section, I appreciated the well-written summary of the main points. I found the take home messages very engaging and think they will work well to invite a skim reader to read the article in more depth.

Reviewer Expertise:

NA

No decision status is available
